# Mechanical Behavior Investigation of Reclaimed Asphalt Aggregate Concrete in a Cold Region

**DOI:** 10.3390/ma14154101

**Published:** 2021-07-23

**Authors:** Wenyuan Xu, Wei Li, Yongcheng Ji

**Affiliations:** School of Civil Engineering, Northeast Forestry University, Harbin 150040, China; xuwenyuan@nefu.edu.cn

**Keywords:** recycled asphalt pavement (RAP) aggregate, mechanical performance, numerical model

## Abstract

Recycled construction and demolition (C&D) waste can reduce the rebuild cost, and is environmentally friendly when recycled asphalt pavement (RAP) aggregate constitutes the main part. This paper investigated the mechanical performance of RAP concrete, and the applicability of RAP in road base layers also was discussed. Several mechanical laboratory tests were selected, including the unconfined compressive-strength, splitting-strength, and compressive-resilience modulus tests. The RAP concrete had a good road performance in a cold region, which was proved by the temperature-shrinkage test, dry-shrinkage test, freeze–thaw-cycle test, and water-stability test. Based on various cement dosages from 3.5% to 5.5% in RAP concrete mix design, three RAP aggregate replacement ratios (30%, 40%, and 50%) were selected to study the variation of mechanical properties with increasing curing time, and the optimal aggregate substitute ratio was determined. A scanning electron microscope (SEM) was used to observe the inner-structure interface between the asphalt binder and cement stone. A numerical model is presented to simulate the RAP compressive strength with respect to the effect of multiple parameters. The research results can provide a technical reference for RAP use in the reconstruction and expansion of low-grade highway projects.

## 1. Introduction

Construction and demolition (C&D) waste management has become a worldwide concern, as up to 600 million tons of waste construction materials are produced each year. Generally, C&D waste mainly contains coarse and fine sand aggregates, aging asphalt, hardened cement hydrate, and other components [1,2,3,4]. C&D waste-recycling research began near the end of the 20th century in the United States, when the Texas Department of Transportation initially investigated the feasibility of using a waste–asphalt mixture in highway construction and maintenance application in 1994. A large number of waste materials were attempted to be used in sub-grade fillers, pavement bases, and other infrastructure construction. The United States saved 4.1 million tons of matrix asphalt and 78 million tons of natural stone in 2018 [5,6]. Similarly, the European Asphalt Pavement Association suggested all its member countries use recycled waste–asphalt materials in 2002. Over 90% of waste–asphalt mixtures have been used for pavement and pavement base materials [7,8,9]. However, with the development of urbanization, about 1.7 billion tons of C&D waste (2019) was generated in China, and the recycling rate is far lower than that in developed countries [10,11,12,13]. Thus, using reclaimed C&D waste in new highway construction is a promising way to solve these imminent issues.

As the largest part of ordinary concrete mixes, the excessive consumption (approximately 26 billion tons per year) of nature aggregate (NA) leads to harmful environmental pollution, and a possible solution to improve the sustainability and cost-effectiveness between C&D and NA needs to be investigated [14,15,16,17]. The reuse of recycled concrete aggregate (RCA) and recycled asphalt pavement (RAP) aggregate in highway rehabilitation has attracted extensive attention in recent years. The RAP aggregate is a mixture of aggregate and bitumen, mainly derived from old asphalt pavement. Due to the asphalt binder, the RAP aggregate has a worse environmental effect and weaker inner bond when compared with RCA.

Existing studies focus on RAP’s material properties and optimal replacement ratio with NA [18,19,20,21,22]. Saeed and Reza [23] evaluated the performance of recycled asphalt mixtures in C&D waste materials, and the optimal binder content was determined. Test results showed that the rutting resistance was effectively improved by 30% in recycled aggregates. Akash et al. [24] used rheological and chemical methods to investigate asphalt binder and mixtures, and various recycling agents were divided into three categories. A novel parameter was developed to predict the effectiveness of various recycling agents. Wojciech [25] evaluated the fatigue life of ASP in an asphalt–concrete mixture, and several lab tests (air-void content, penetration, stiffness) were made to evaluate the mixture’s mechanistic performance. A French method was presented to calculate the mixture’s fatigue life. Juntao et al. [26] tested an eight-year recycled asphalt mixture bound by emulsion, and the long-term performance and interface microstructure were discussed. Test results showed that the tensile strengths and creep deformation met the performance requirement. Hassan [27] studied the behavior of reclaimed asphalt pavement (RAP) aggregate concrete, and various mixtures (natural aggregate, reclaimed coarse aggregate, reclaimed coarse and fine aggregate, and reclaimed coarse aggregate with 30% fly ash) were selected to find the optimum performance. Research results showed that the RAP aggregate partially reduced the concrete’s mechanical performance (compression and tensile strength). However, the properties of ductility and microstructure were improved due to the effect of fly ash and RAP aggregate. Papakonstantinou [28] investigated the performance of recycled asphalt pavement (RAP) aggregate use in Portland cement concrete (PCC). Five weight percentages (5%, 7.5%, 10%, 12.5%, and 15%) of RAP aggregates were used in concrete mix design, and their mechanical performance was examined. The test results found that the compression strength and elastic modulus had a negative relation with increasing RAP ratio, and all mixtures met the requirements for road performance. Zaumanis et al. [29] proposed a performance-based design method to solve the asphalt mix design procedures, and the key parameters were determined to improve the asphalt mixture’s performance.

Other studies investigated RAP concrete applications, including new asphalt pavement, base layers, structural members, and so on [30,31]. Giulia et al. [32] summarized the development of reclaimed asphalt pavement (RAP) material used in new asphalt construction, and the effect of RAP content was discussed. Abdulgazi [33] discussed the potential utilization of construction demolition waste (CDW) using in hot-mix asphalt pavements, and alternative CDW technical specifications and guidelines were presented that mainly considered safety, tolerability, and efficacy. Fawaz et al. [34] summarized the current knowledge about reclaimed asphalt pavement (RAP) and recycled asphalt shingles (RAS) in the United States, and the current and future challenges for reclaimed asphalt utilization were presented. Sharareh et al. [35] evaluated the effects of recycled asphalt shingles (RAS) on pavement performance. A cost analysis was conducted to assess the life-cycle cost of asphalt pavements constructed with RAS. Then, a mix with 5% post-consumer waste shingles and recycled asphalt had the lowest cost over the pavement’s service life. Nasim [36] investigated axial compression behavior of concrete columns concerning four types of aggregates (NA, RCA, RAP, and RCA-RAP). Reclaimed aggregate replacement ratios from 20% to 100% also were considered. Test results indicated that the ultimate failure load had a decreasing trend with the increase of the reclaimed aggregate substitute ratio, but the structure’s safety was proved.

This study’s main objective was to investigate the mechanical performance of RAP concrete, and several mechanical and physical laboratory tests were selected to evaluate its road performance in a cold region. Based on the various cement dosages, from 3.5% to 5.5% in the RAP concrete mix design, three recycled aggregate mixture contents (30%, 40%, and 50%) were selected to study the variation of mechanical behavior concerning different curing times, and the optimal RAP aggregate-substitute ratio was determined. A scanning electron microscope (SEM) was used to observe the inner-structure interface between the asphalt binder and cement stone. A numerical model was created to simulate the RAP’s compressive strength with respect to the effect of multiple parameters. The research results can provide a technical reference for RAP use in the reconstruction and expansion of low-grade highway projects.

## 2. Experimental Programs

### 2.1. Reclaimed Asphalt Mixture Sieve Analysis and Mix Design

**Figure 1:**
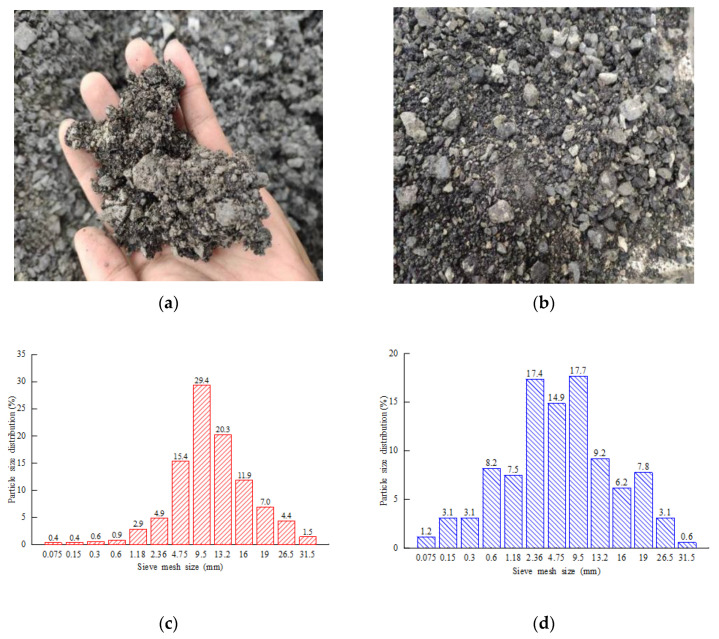
Reclaimed asphalt mixture: (**a**) coarse aggregate; (**b**) fine aggregate; (**c**) untreated. (**d**) Extracted RAP particle-size distribution (PSD).

Typically, asphalt pavement needs a high temperature for constant construction, and a thermal aging action occurs. In addition, the aging effect will be accelerated by light, weathering, snow/ice cover, rainwater penetration, and traffic load. In this paper, the reclaimed asphalt mixture came from the reconstruction and expansion of the National Dana Highway. The pavement structure was built in 2005, and consisted of 10 cm-thick asphalt concrete pavement. Due to the aging effect, the mechanical performance of the existing asphalt pavement cannot meet the serviceability. Therefore, the more extensive mixture was broken manually and then crushed with a small jaw crusher. Figure 1a,b show the RAP coarse and fine mixtures after being crushed by the jaw crusher, and some of them are clustered structures because of asphalt bonding. In addition, it can be observed that the asphalt and aggregate were separated due to sunlight weathering and traffic load. In order to reduce the difference of gradation composition, the RAP aggregate was sieved with an asphalt mixture centrifugal-extraction apparatus, as shown in Figure 1c,d.

Table 1 shows the physical properties of the RAP and NA aggregates. The RAP aggregate exerts a higher void content and lowers apparent density, and it can be attributed to uneven surfaces and microcracks generated during the RAP crushing process. In addition, the existing asphalt and cement binder lead to the enlarging of the inner air void, eventually causing a noticeable increase in water absorption for RAP.

Considered with the various cement dosages from 3.5% to 5.5%, three recycled aggregate mixture contents (30%, 40%, and 50%) were selected in the RAP concrete mix design. The F30-S3.5 specimen represents 30% RAP and 70% NA, and the cement dosage is 3.5%. A total of nine combination specimen types were used, and their optimum water content and maximum dry densities are shown in Table 2.

### 2.2. Axial Compressive-Strength Test

**Figure 2:**
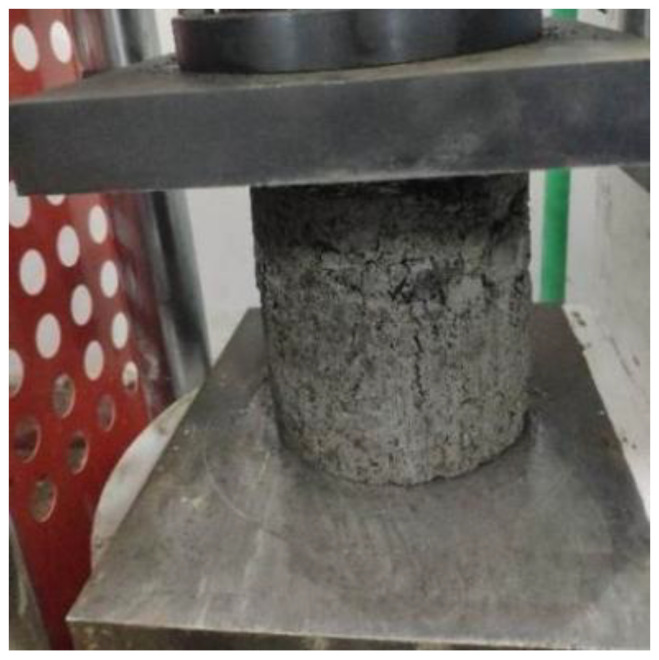
Axial compressive-strength test.

According to the Highway Engineering Inorganic Binder Stability Materials Code (JTG E51-2009), a cylinder size with 150 mm diameter by 150 mm depth was selected. Its compression test setup is shown in Figure 2. Two variable parameters were considered in the experimental test: a reclaimed aggregate replacement ratio from 30% to 50%, and cement dosage from 3.5% to 5.5%. The specimens’ compressive strengths were compared with respect to various curing times, from 7 to 90 days.

### 2.3. Splitting Tensile Strength Test

**Figure 3:**
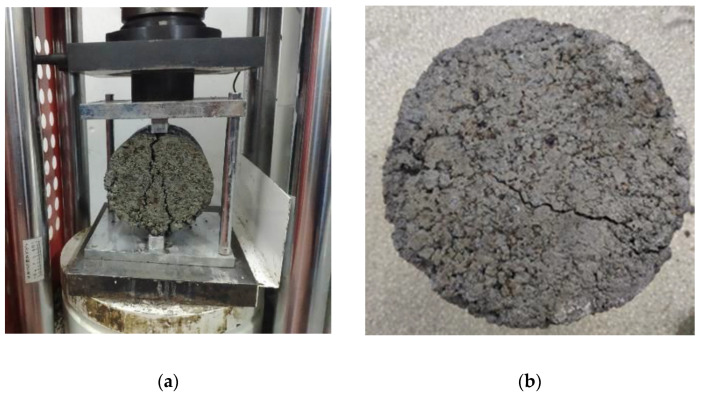
Splitting tensile-strength test: (**a**) test setup; (**b**) test specimen.

According to Highway Inorganic Bond Stabilization Materials Splitting Test Method (JTG E51-2009 T0806-1994), specimens with different cement dosages and reclaimed asphalt mixtures were selected for 28- and 90-day curing times. Figure 3 shows the splitting-test setup and one typical test specimen.

**Figure 4:**
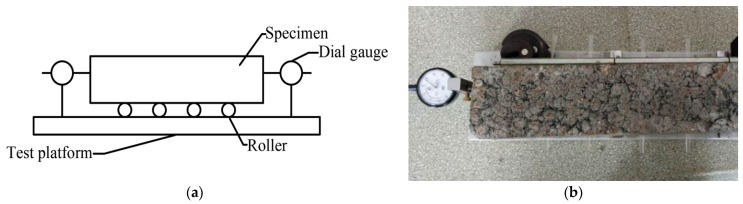
Compressive-resilience modulus test: (**a**) test setup; (**b**) test specimen.

Similar to the splitting-strength test, specimens with two different curing times (28 and 90 days) were selected to test their compressive resilience moduli, and the relationship between multi-stage loading and their deformation was considered, as shown in Equation (1):

(1) $$E_{c}=\frac{\mathrm{ph}}{l}$$

where E_c_ is compressive resilience modulus (MPa); p is unit pressure (MPa); h is the specimen height (mm); and *l* is the resilient deformation (mm). Figure 4 shows the compressive-resilience modulus test setup and one typical test specimen.

### 2.4. Dry- and Temperature-Shrinkage Tests

**Figure 5:**
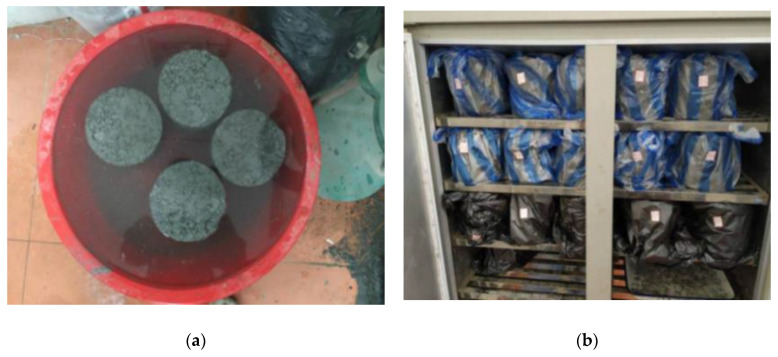
Shrinkage test: (**a**) water immersion; (**b**) temperature-controlled cabinet.

According to Highway Inorganic Bond Stabilization Materials Splitting Test Method (T0854-2009), water-loss rate and dry-shrinkage strain were selected to investigate the ability of deformation resistance under the presence of pore water. The water-loss rate was calculated with the mass variation when subjected to immersion and dry conditions, as shown in Figure 5a. The dry-shrinkage strain was measured with a micrometer gauge, and the monitor period was divided into two interval times, which was recorded daily during the first week, and extended from 2 to up to 27 days.

Similarly, the temperature-shrinkage strain was measured with a micrometer gauge, and a lab temperature-controlled cabinet was required. Based on the on-site monitored data in Huma town (China), temperatures of 14.9 °C~18.7 °C in summer and −30.2~−20.1 °C in winter are observed. Thus, the temperature-shrinkage measuring zone was determined to be from 20 °C to −30 °C with an interval of 10 °C, as shown in Figure 5b.

### 2.5. Freeze-Thaw Cycle and Water-Stability Test

**Figure 6:**
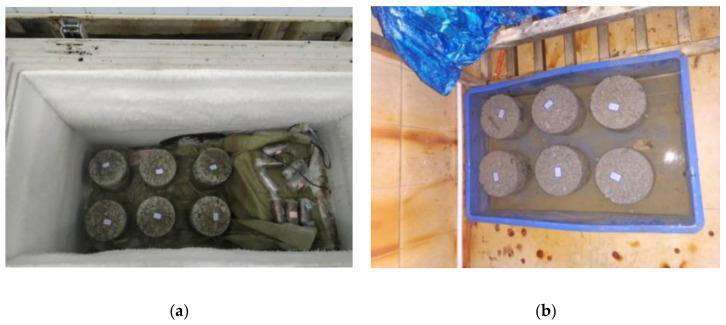
Freeze–thaw cycle and water-stability test: (**a**) freeze–thaw test; (**b**) water immersion.

Most road-base materials are macroporous, and strength loss frequently occurs during the freeze–thaw cycle. The specimens’ mass and strength loss needed to be investigated through a freeze–thaw test in the seasonally frozen ground region. The 28-day and 90-day specimens were selected for the freeze–thaw test, and 10 cycles were used (frozen for 16 h at −18 °C and thawed for 8 h in 20 °C = one cycle), as shown in Figure 6a.

The water-stability test examined the waterproof performance or water resistance of the road sub-base layer. For example, one water-stability cycle included initial immersion in water for 1 d and air-drying for 2 d, and then soaking in water for 1 d and air-drying for 2 d, as shown in Figure 6b. After five cycles, the compressive strength of the specimen was measured and compared with the specimen without immersion, and the calculation formula is shown in Equation (2):

(2) $$S=\frac{R_{\mathrm{sc}}}{R_{c}}\;\times \;100$$

where S is the coefficient of water stability; R_sc_ is the specimen strength after five cycles; and R_c_ is specimen strength without cyclic immersion.

## 3. Experimental Test Results

### 3.1. Sieve Analysis

**Figure 7:**
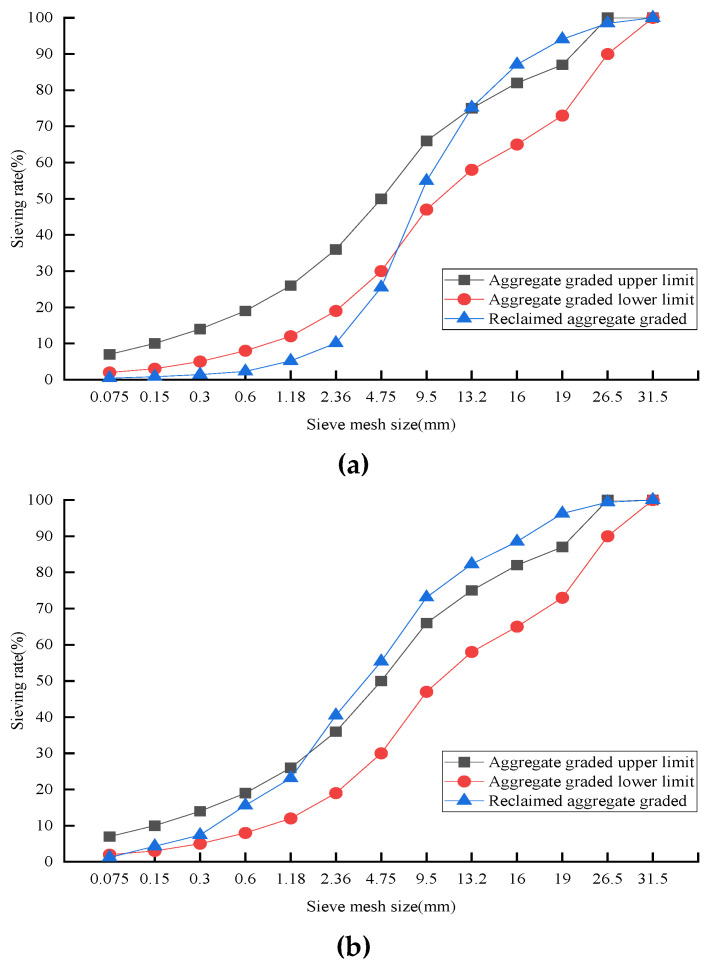
Reclaimed asphalt sieve analysis: (**a**) untreated RAP mixture; (**b**) extracted RAP mixture.

Based on the centrifugal separation method in the Highway Engineering Asphalt and Asphalt Mixture code (JTG E20-2011), a centrifugal extraction apparatus was used to extract the waste–asphalt mixture. The 7- and 10-year RAP samples were selected, and softening point and penetration were tested to analyze the aging effects. With the increase of service life, penetration showed a decreasing trend, but the softening point showed the opposite (penetration: 65.1% for 7 years vs. 38.3% for 10 years, and softening point: 11.6% vs. 41.0%). It was observed that the coarse aggregate occupied a high portion of the waste–asphalt mixture, and a particle size below 2.36 mm accounted for about 10% of the total mass. This can be attributed to the fine aggregate bonding with asphalt to form larger particles, as shown in Figure 7a. The performance of asphalt deteriorated with time, and the sieve results of the extracted mixture are shown in Figure 7b. Compared with the untreated asphalt mixture, the fine aggregate particle-size distribution showed an apparent improvement between the upper and lower limits. It can be concluded that the untreated RAP aggregate required the extraction process before casting concrete to meet the gradation composition requirement.

### 3.2. Axial Compressive-Strength Tests

**Figure 8:**
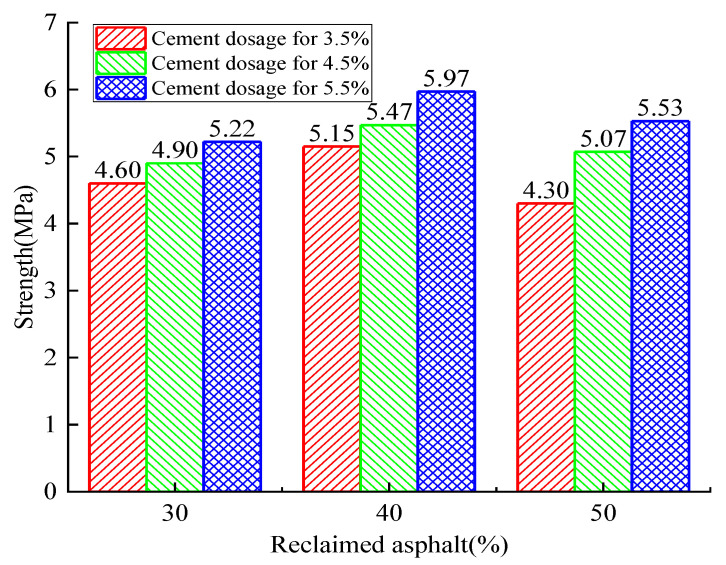
Analysis of the 7-day axial compressive-strength test with variation of cement dosage and RAP.

**Figure 9:**
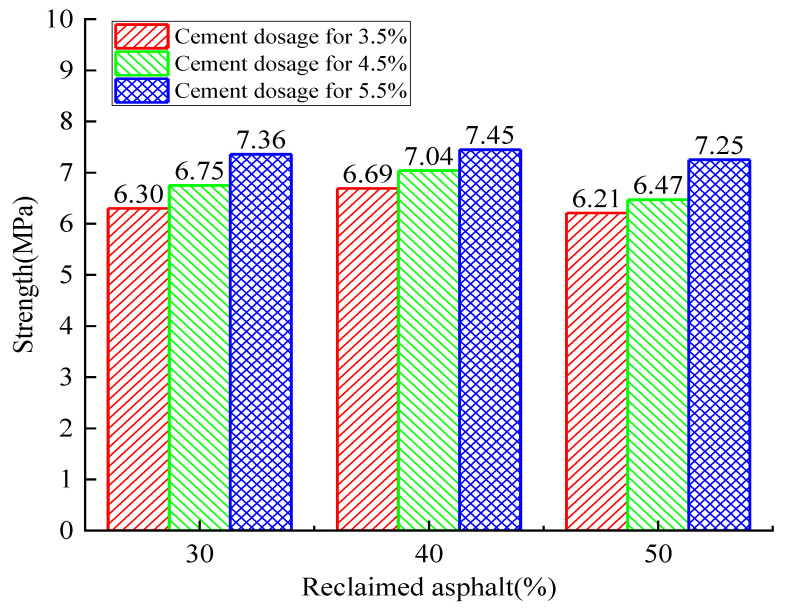
Analysis of the 28-day axial compressive-strength test with variation of cement dosage and RAP.

**Figure 10:**
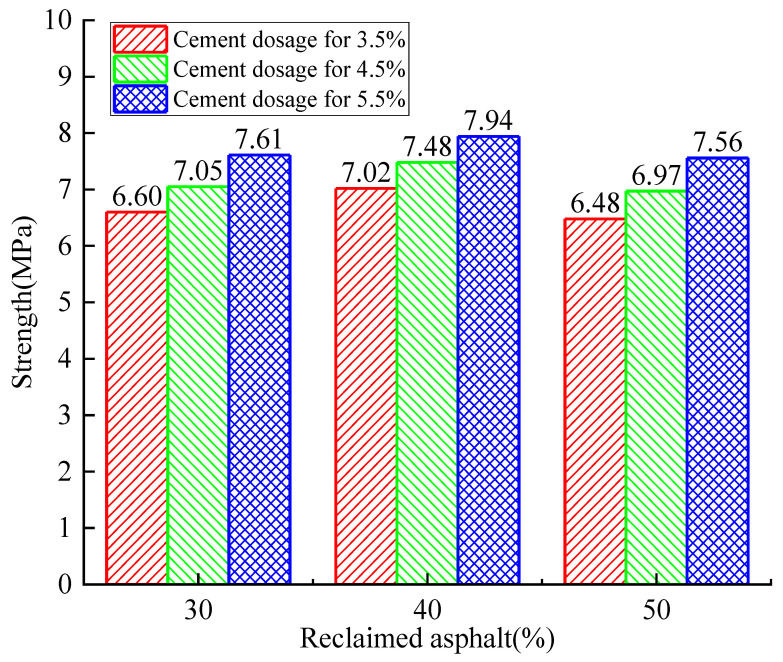
Analysis of the 90-day axial compressive-strength test with variation of cement dosage and RAP.

Figure 8, Figure 9 and Figure 10 show the RAP axial compressive-strength variation when subjected to cement dosages from 3.5% to 5.5%. It was observed that the cement dosage had a positive effect on axial strength, and the optimal cement dosage was 5.5%. Similarly, the optimal RAP substitute ratio was determined to be 40%.

**Figure 11:**
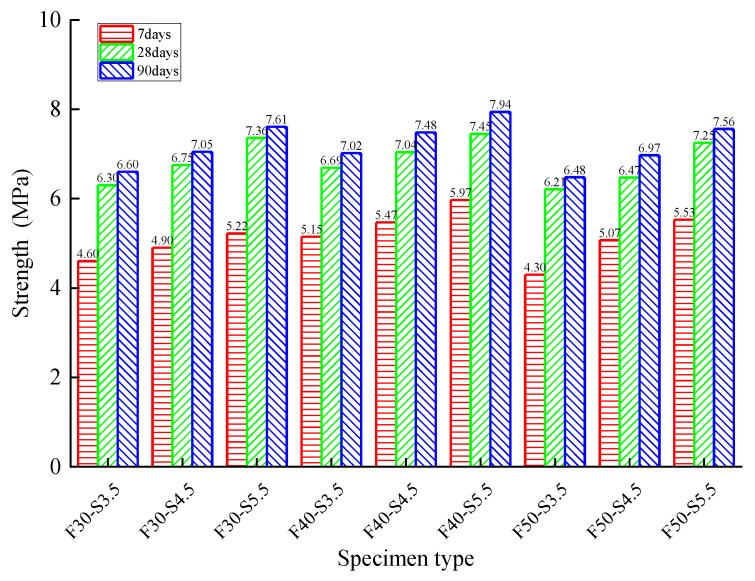
The relationship between axial compressive strength and curing time for various specimens.

The relationship between axial compressive strength and curing time is shown in Figure 11. When the RAP replacement ratio was increased from 30% to 40%, the axial compressive strength increased by 1.22% to 14.37% as the cement dosage remained constant. On the contrary, the axial compressive strength decreased by 2.68% to 16.50% when the RAP replacement ratio reached 50%. Therefore, it can be concluded that the axial strength increases initially and decreases afterward with the increase of the RAP replacement ratio. However, the axial compressive strength exerted a continuous positive effect (4.19~17.91% for 4.5% and 5.82~12.06% for 5.5%) with the increase of cement dosage when the RAP content remained constant. All these observations show that a critical RAP substitute ratio and cement dosage exist to achieve the maximum RAP axial strength. The cost efficiency and environmental friendliness also need to be balanced.

Similarly, the compressive strength showed an increasing trend with curing time no matter the amount of cement dosage and RAP (24.79~40.99% from 7 to 28 days, and 4.28~7.73% from 28 to 90 days). The increasing trend turning flat can be explained by the hydration reaction gradually finishing.

### 3.3. Splitting Tensile-Strength Tests

**Figure 12:**
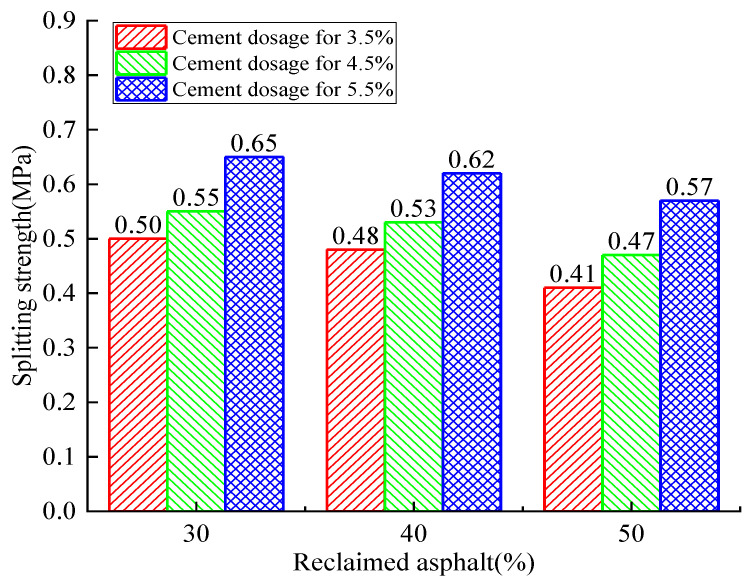
Analysis of the 28-day reclaimed asphalt splitting tensile-strength test with variation of cement dosage and RAP.

The 28-day specimen’s splitting-strength relationship between RAP and cement dosage is shown in Figure 12. It can be observed that the RAP specimen’s splitting strength showed an upward trend with the increase of cement dosage. For the different RAP replacement ratios, an increasing trend was observed for all cement dosage cases, but the growth rate and maximum splitting strength varied. The maximum growth rate was 21.4% when subjected to the 5.5% cement dosage and 50% RAP replacement ratio. For the 30% RAP replacement ratio and 5.5% cement dosage, the maximum splitting strength reached 0.65 MPa. Similarly, the splitting strength showed a slowly decreasing trend with an increase of the reclaimed asphalt mixture replacement ratio. The higher cement dosage showed a better splitting resistance (16.21% for 3.5% vs. 9.1% for 5.5%). The lower RAP replacement ratio showed a higher splitting strength. It can be concluded that the RAP aggregate substitute NA had a negative influence on concrete’s splitting tensile strength, and the increasing of cement dosage effectively relieved this strength degradation.

**Figure 13:**
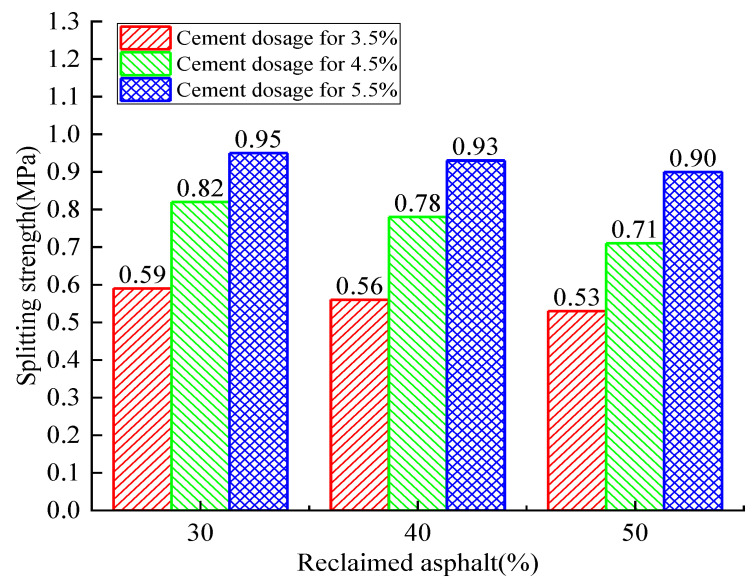
Analysis of the 90-day reclaimed asphalt splitting tensile-strength test with variation of cement dosage and RAP.

For 90 days of curing time, the splitting strength relationship between cement dosage and reclaimed asphalt replacement ratio is shown in Figure 13. The 90-day splitting strength had a similar increasing trend to that of 28 days, but a higher splitting strength and growth rate were observed. For the 30% reclaimed asphalt mixture, the highest growth rate of 39.1% was observed when compared with cement dosage from 3.5% to 4.5%. Therefore, the maximum splitting strength was 0.95 MPa for the 30% reclaimed asphalt mixture and 5.5% cement dosage.

**Figure 14:**
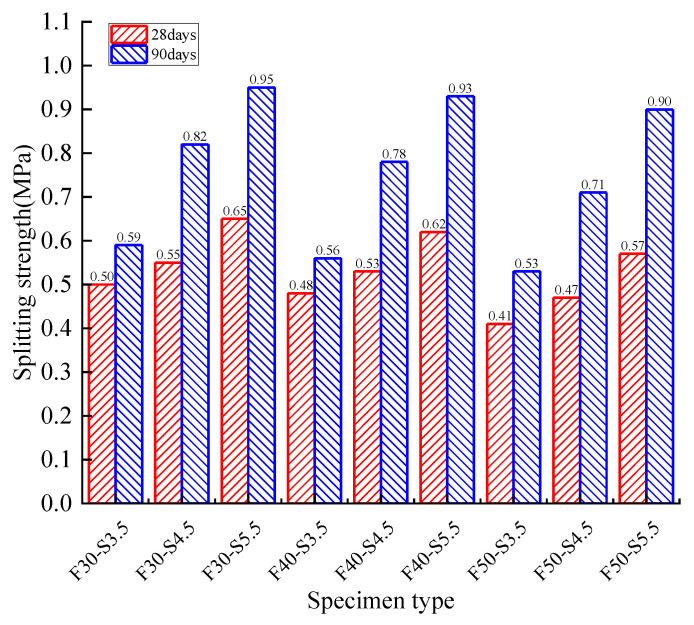
Reclaimed asphalt splitting tensile strength with respect to curing time.

Figure 14 shows the 90-day splitting strength concerning the variation of reclaimed asphalt mixture and cement dosage. The splitting strength showed a descending trend with the increase of reclaimed asphalt. The 4.5% cement dosage and 50% reclaimed asphalt exerted the highest strength-loss rate (8.8%). Compared with 28 days, specimens’ splitting strength growth rate showed an increasing trend, up to 44.7% for 90 days curing time.

It can be concluded that the cement dosage had a positive effect on splitting strength when the reclaimed asphalt mixture remained constant. However, reclaimed asphalt had a negative effect as the cement dosage was constant. Therefore, the splitting strength was a positively correlated function of curing time if the cement dosage and reclaimed asphalt remained constant.

**Figure 15:**
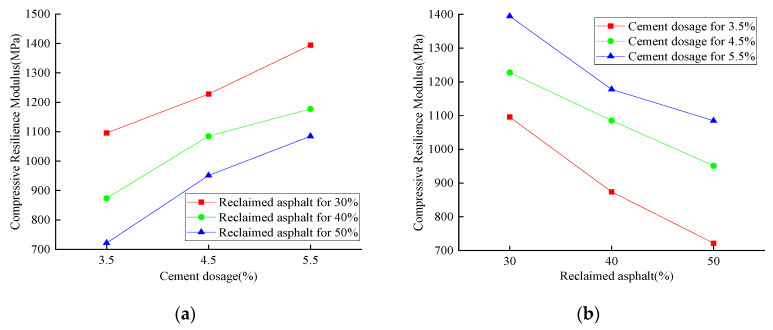
Results for the 28-day compressive-resilience modulus test: (**a**) cement dosage variation; (**b**) RAP replacement ratio.

**Figure 16:**
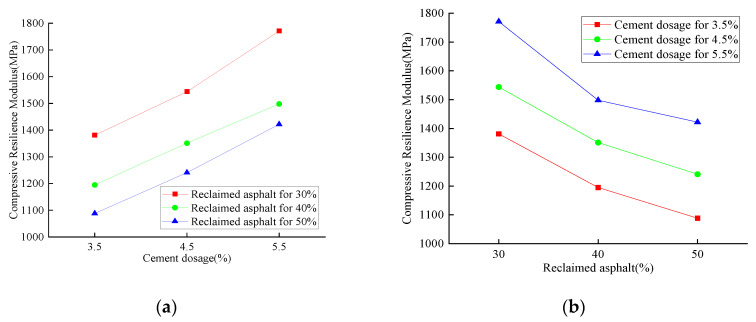
Analysis of the 90-day compressive-resilience modulus test: (**a**) cement dosage variation; (**b**) RAP replacement ratio.

**Figure 17:**
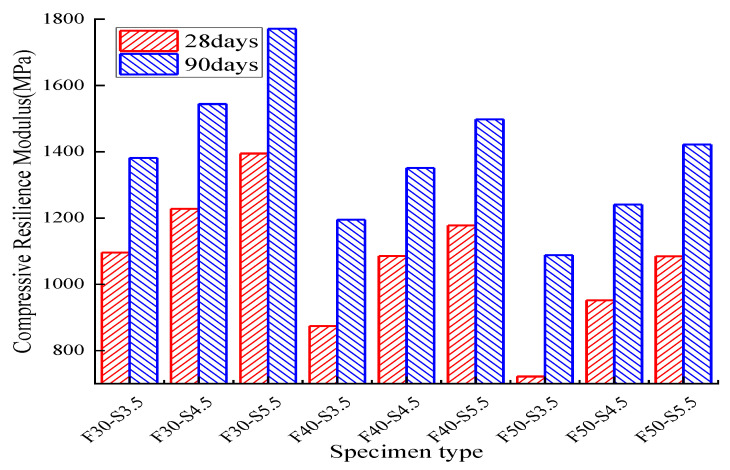
Compressive-resilience modulus test results with respect to curing time.

The compressive resilience modulus of reclaimed asphalt with various cement dosages is shown in Figure 15, Figure 16 and Figure 17. The compressive resilience modulus was positively correlated with cement dosage while negatively affecting the RAP replacement ratio, no matter the curing-time variation. For 28 days of curing time, the minimum compressive resilience modulus was 721.8 MPa (3.5% cement dosage and 50% reclaimed asphalt), and the maximum value was 1394.7 MPa (5.5% cement dosage and 30% reclaimed asphalt). For 3.5% cement dosage, the maximum compressive resilience modulus loss was 20.2% when the RAP content was increased from 30% to 40%. With an increased curing time of up to 90 days, the compressive resilience modulus showed an increasing trend, and the maximum value reached 1771.2 MPa. It was concluded that the increase of compressive resilience modulus mainly depended on the concrete curing time and cement dosage. However, the RAP had a significant negative effect when compared with NA.

### 3.4. Dry- and Temperature-Shrinkage Tests

**Figure 18:**
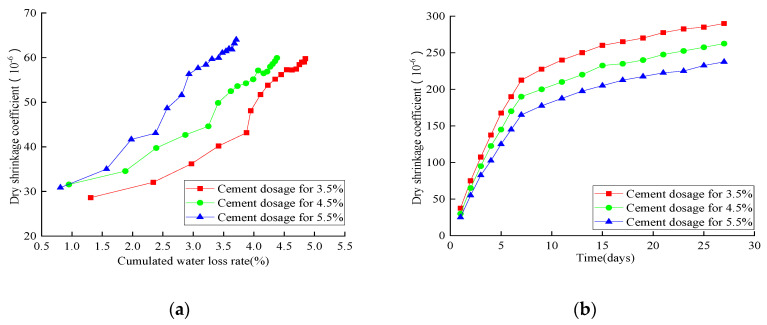
Analysis of the dry-shrinkage test: (**a**) dry-shrinkage coefficient related to water-loss rate; (**b**) dry-shrinkage strain related to time.

Figure 18 shows the relationship between the dry-shrinkage coefficient and water-loss rate. With the increase of water-loss rate, the drying-shrinkage coefficient showed a similar increasing trend when the cement dosage was increased from 3.5% to 5.5%. Three increase stages were observed, which included the slowly ascending stage (0–3.4% for 3.5%, 0–3.2% for 4.5%, and 0–2.6% for 5.5%), rapid-growth stage (3.4–4.5% for 3.5%, 3.2–4% for 4.5%, and 2.6–3.4% for 5.5%), and steady stage. Compared with other cases, the 5.5% cement dosage had the maximum shrinkage coefficient and minimum water-loss rate. With the increase of curing time, the drying-shrinkage strain showed a fast and then slow increasing trend. The drying-shrinkage coefficient gradually increased with the decrease of cement dosage—1.15 times for 4.5% and 1.25 times for 5.5%. The drying-shrinkage strain gradually increased with the increase of water-loss rate, and it also had a positive effect with cement dosage on constant water-loss rate. Two increase zones were observed, which included a slowly ascending zone (0–3.4% for 3.5%, 0–3.2% for 4.5%, and 0–2.6% for 5.5%) and a rapid growth zone (larger than 3.4% for 3.5%, larger than 3.2% for 4.5%, and larger than 2.6% for 5.5%). All these observations showed that the higher cement dosage facilitated an adequate cement hydration reaction, leading to higher shrinkage. Beyond that, more cement particles filled the RAP aggregate void and occupied the space that originally belonged to water.

**Figure 19:**
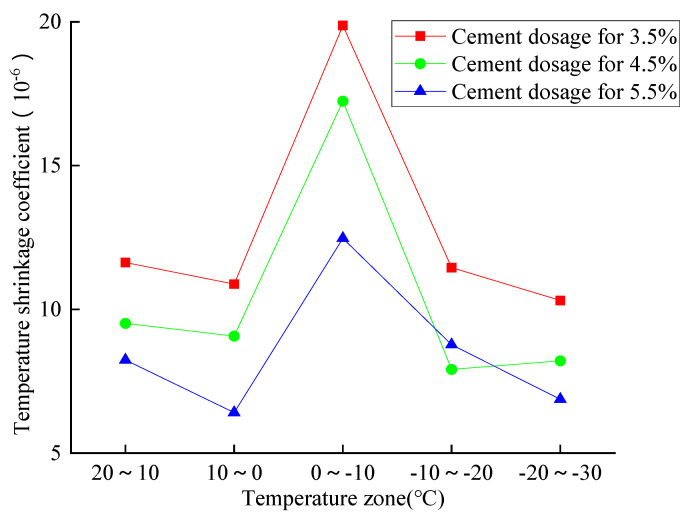
Analysis of the temperature shrinkage coefficient.

The temperature shrinkage data related to 40% reclaimed asphalt and cement dosages from 3.5% to 5.5% are summarized in Figure 19. The temperature-shrinkage coefficient showed a gradually decreasing trend with an increase of cement dosage, and all shrinkage values were located between 6.41 × 10^−6^ and 19.9 × 10^−6^. The 3.5% cement dosage exerted the maximum temperature shrinkage coefficient, and the sharply ascend zone occurred between 0 °C and −10 °C. All the observations can be attributed to the pore water in specimens causing a freezing volume expansion between 0 °C and −10 °C, and a slight shrinkage coefficient was seen in other temperature zones.

### 3.5. Freeze-Thaw Cycle and Water-Stability Tests

**Figure 20:**
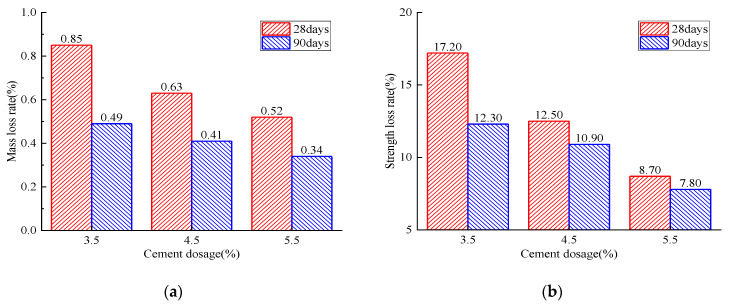
Analysis of the freeze–thaw-cycle test: (**a**) mass loss rate; (**b**) strength loss rate.

With the increase of curing time from 28 to 90 days, both the mass and strength loss rate gradually decreased the exact cement dosage, as shown in Figure 20. For the same curing time, the increased cement dosage had a positive effect on maintaining the strength and mass of the RAP specimen. All these observations imply that the increased curing time and cement dosage effectively reduced the RAP specimen’s internal porosity, which mainly depended on the degree of cement hydration reaction. It can be concluded that the higher cement dosage and longer curing time effectively improved the RAP frost resistance.

**Figure 21:**
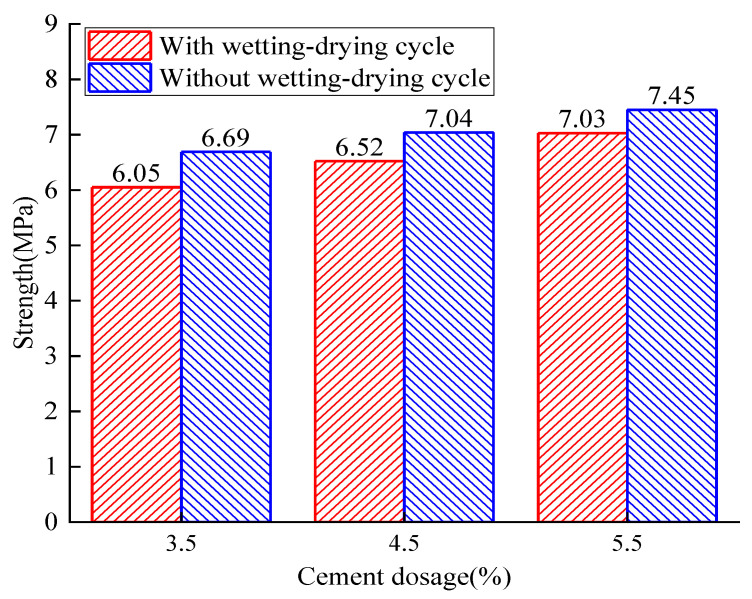
Analysis of the water-stability test.

For specimens with 40% reclaimed asphalt and various cement dosages from 3.5% to 5.5%, the compressive strength related to dry–wet cycles (with and without) is summarized in Figure 21. It can be observed that the dry–wet cycles reduced the specimens’ compressive strength, and the water-stability coefficient exerted a good water resistance with an increase of cement dosage.

### 3.6. Microcosmic Analysis

Unlike cement stone and aggregate direct adsorption, an asphalt layer exists between cement stone and aggregate in reclaimed asphalt specimens. In order to observe the interface between cement and asphalt, the scanning electron microscope (SEM) method is used to investigate the influence of existing asphalt. The asphalt sample was in a solid state in a reclaimed asphalt mixture and was not heated during recycling.

**Figure 22:**
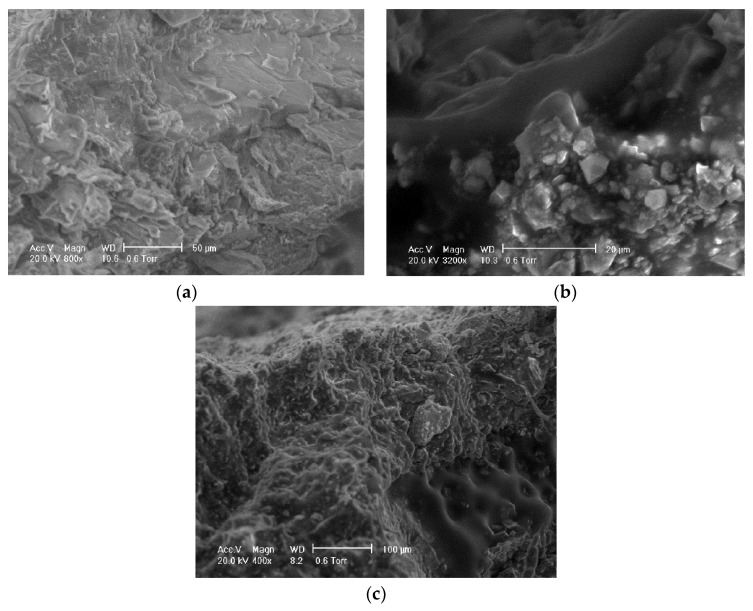
Scanning electron microscope (SEM) method: (**a**) cement stone; (**b**) interface between cement stone and asphalt; (**c**) bituminous mixture particles bond to cement.

Figure 22a shows the morphology diagram of cement stone in the reclaimed asphalt mixture, and the observation area is not in contact with reclaimed asphalt. Compared with traditional cement stabilized materials, the RAP surface was uneven and angular, validated by higher void content as shown in Table 1. Figure 22b shows the interface between cement stone and asphalt in the reclaimed asphalt mixture. It can be observed that the asphalt was effectively filled with the porosity within the cement mixture, forming embedded connection strength. Due to the crushing process of RAP aggregate, the cement stone and asphalt were bonded with an irregular dividing line, and a concave and convex shape was observed. Adhesion partially retarded the hydration reaction between the cement mortar and aggregate, which caused the lower strength generation. Figure 22c shows the asphalt particle embedded in cement stone, and some small particles are also absorbed on cement stone due to crushing and mixing. Many microholes appeared on the surface of bituminous mixture particles. This can be explained by the higher water absorption than for the NA aggregate (Table 1).

## 4. Numerical Simulation Analysis

**Figure 23:**
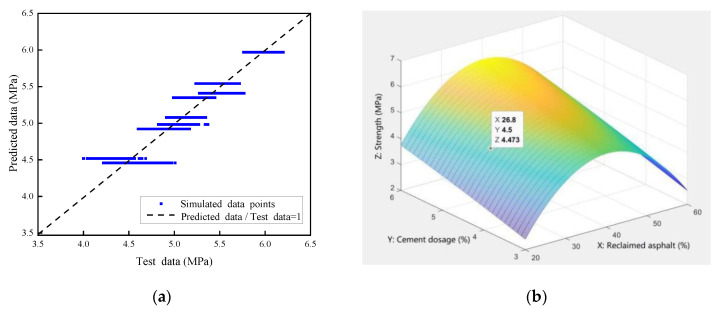
Numerical simulation of compressive strength: (**a**) Monte Carlo simulation; (**b**) 3D diagram of the nonlinear strength-fitting equation.

The 7-day compressive-strength test data (Figure 8) was selected to establish a numerical analysis model, which was mainly used to predict the variation law of the reclaimed asphalt mixture’s compressive strength with respect to the influence of multiple factors. Two independent variables were considered in the presented numerical analysis model: reclaimed asphalt replacement ratio (30%, 40%, and 50%) and cement dosage (3.5%, 4.5%, and 5.5%). Due to the limited test data, the Monte Carlo simulation method was used to expand the sample number to a sizeable, reasonable size (4500 samples). The simulation data distribution was assumed to be a normal distribution, and the data boundary was selected for mean value ± standard deviation, as shown in Figure 23a. Compared with the relative errors, the predicted points had good agreement with the tested values (R^2^ = 0.908), which met the accuracy requirements of the model prediction analysis. A bivariate nonlinear strength-fitting equation is presented based on the nonlinear regression analysis method, as shown in Equation (3):

Z = −6.384 + 0.475X + 0.604Y − 0.0059X^2^ − 0.0176Y^2^ (20% ≤ X ≤ 60%, 3% ≤ Y ≤ 6%)(3)

where X is the reclaimed asphalt mixture content (%); Y is cement dosage (%); and Z is the 7-day compressive strength (MPa).

In order to exhibit the relationship among variables in the bivariate nonlinear strength equations, a three-dimensional effect diagram was constructed in MATLAB. Figure 23b shows the three-dimensional surface related to X (content of reclaimed asphalt mixture), Y (cement dosage), and Z (7-day compressive strength). For example, a point in the figure (X 26.8; Y 4.5; Z 4.473) represents a predicted 7-day compressive strength of 4.473 MPa when subjected to the reclaimed asphalt mixture content of 26.8% and a cement dosage of 4.5%. It can be concluded that the 7-day compressive strength can be predicted in the presented numerical model when subjected to various cement dosages and reclaimed asphalt mixture, and vice versa.

**Figure 24:**
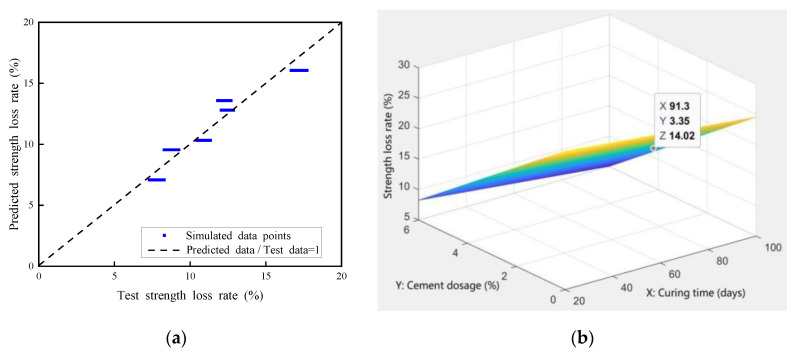
Numerical simulation of compressive-strength-loss rate: (**a**) Monte Carlo simulation; (**b**) 3D diagram of nonlinear strength-loss fitting equation.

Similarly, considering the effect of freeze–thaw cycles, a revised numerical analysis model was established to predict the strength reduction of the reclaimed asphalt mixture. For example, two variables were considered for the 40% reclaimed asphalt mixture content: curing time (28 and 90 days) and cement dosage (3.5%, 4.5%, and 5.5%). Due to test data being limited, the Monte Carlo method was used to simulate 3000 samples, and the data-distribution type was assumed to be a normal distribution. Figure 24a shows that the simulated data points were in good agreement with the test values (R^2^ = 0.915), which met the accuracy requirements of the model prediction analysis. The nonlinear regression analysis method was used to create a bivariate nonlinear strength-fitting equation, as shown in Equation (4):

Z = 28.5561 − 0.0398275X − 3.25298Y (20 ≤ X ≤ 100, 0% < Y ≤ 6%)(4)

where X is the curing time of reclaimed asphalt mixture; Y is cement dosage (%); and Z is the compressive strength loss rate (%) of reclaimed asphalt mixture.

Figure 24b shows the relationship among X (curing time), Y (cement dosage), and Z (compressive strength loss rate) in the 3D model. For example, a point was randomly selected in the figure (X 91.3; Y 3.35; Z 14.02), representing the predicted value of compressive strength loss rate of 14.02% for 91.3 days curing time and 3.35% cement dosage. It can be concluded that the presented numerical model could effectively predict the reclaimed asphalt mixture strength reduction when subjected to the freeze–thaw effect.

**Figure 25:**
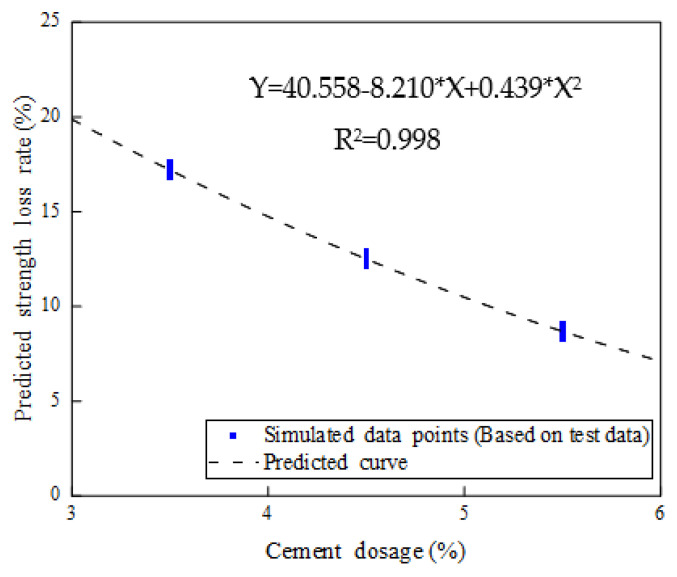
Nonlinear strength-loss-rate fitting equation with respect to freezing-damage effects.

A simplified numerical model was created for practical engineering applications when considering the relationship between cement dosage and 28-day strength-loss rate. The nonlinear regression method was used to build the strength-degradation equation (Equation (5)), which included 1500 data points by Monte Carlo simulation:

Y= 40.558 − 8.210X + 0.439X^2^ (3% ≤ X ≤ 6%) (5)

where X is the cement dosage (%), and Y is the 28-day compressive-strength-loss rate (%) under the freeze–thaw effect. Compared with the relative error between the predicted value and the tested value, the 1500 simulated data points showed a good consistency with the measured value (R^2^ = 0.998), which could meet the accuracy requirements of the model-prediction analysis, as shown in Figure 25.

**Figure 26:**
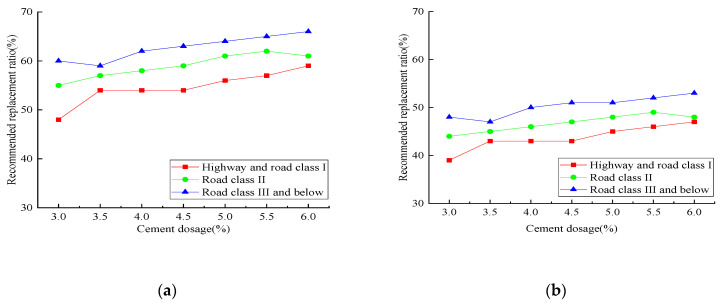
The maximum replacement ratio of reclaimed asphalt with respect to various cement dosage: (**a**) with the freeze–thaw effect; (**b**) without the freeze–thaw effect.

In order to facilitate data selection for the construction technicians, the maximum replacement ratio of reclaimed asphalt is presented for various cement dosages, considering both with and without the freeze–thaw effect. Figure 26 shows the maximum replacement ratio of reclaimed asphalt with respect to various cement dosages. There was an apparent descending trend when considering the freeze–thaw effect, and the increasing cement dosage effectively improved the replacement ratio. It can be concluded that the maximum replacement ratio of the reclaimed asphalt mixture can be selected with different cement dosages. For specific cement dosages, the linear interpolation method can be used to obtain the maximum replacement ratio.

## 5. Conclusions

This paper investigated the mechanical performance of reclaimed asphalt mixtures. More specifically, mechanical properties such as compressive strength, splitting strength, compressive modulus of resilience, were examined, as well as the results of freeze–thaw cycling tests. The main conclusions were as follows:

According to the axial compressive and splitting tensile-strength tests, critical RAP substitute ratios (30%) and cement dosage (5.5%) exist to achieve the maximum mechanical performance. However, the cost efficiency and environmental friendliness also need to be balanced. It can be concluded that the RAP aggregate substitute NA had a negative influence on concrete compression and splitting tensile strength, and the increasing of cement dosage effectively relieved this strength degradation.

It seems that the higher cement dosage represented an adequate cement hydration reaction through dry- and temperature-shrinkage tests, which led to higher shrinkage occurring. Beyond that, a noticeable temperature-shrinkage-increase zone (between 0 °C and −10 °C) was observed due to the pore water freezing and causing a volume-expansion effect.

The 5.5% cement dosage and 90 days of curing time exerted the most robust RAP frost resistance in the freeze–thaw-cycle and water-stability tests. Therefore, it can be concluded that the increased curing time and cement dosage effectively reduced the RAP specimen’s internal porosity, and mainly depended on the degree of the cement hydration reaction.

In the scanning electron microscope (SEM) analysis, an irregular dividing line between the cement stone and asphalt was observed for the RAP aggregate. The concave and convex surfaces partially retarded the hydration reaction between the cement mortar and aggregate, which caused lower strength generation and higher water absorption than NA.

A nonlinear compression-strength regression equation was presented using RAP content and cement dosage through a Monte Carlo numerical-simulation method. Considered with different highway-grade requirements, the corresponding RAP replacement ratio and cement dosage limits were determined.

## Figures and Tables

**Table 1:** Physical properties of aggregates.

Aggregate Type	Size (mm)	Apparent Density (g/cm³)	Water Absorption (%)	Void Content (%)
RAP	5–10	2.618	7.41	44.5
	10–20	2.666	5.31	45.3
NA	5–10	2.721	0.91	42
	10–20	2.719	0.45	43

**Table 2:** RAP concrete mix design.

No.	Optimum Water Content (%)	Maximum Dry Density (g/cm^3^)
F30-S3.5	5.24	2.25
F40-S3.5	4.93	2.29
F50-S3.5	4.90	2.21
F30-S4.5	5.25	2.29
F40-S4.5	5.05	2.31
F50-S4.5	5.04	2.22
F30-S5.5	5.30	2.31
F40-S5.5	5.22	2.33
F50-S5.5	5.21	2.25

## Data Availability

Not applicable.
